# Correction: Non-invasive ventilation with pursed lips breathing mode for patients with COPD and hypercapnic respiratory failure: A retrospective analysis

**DOI:** 10.1371/journal.pone.0247693

**Published:** 2021-02-19

**Authors:** Christoph Jünger, Maja Reimann, Lenka Krabbe, Karoline I. Gaede, Christoph Lange, Christian Herzmann, Stephan Rüller

In Fig 4, panel B does not appear. In Fig 5, panels B and C do not appear. Please see the correct Figs 4 and 5 here.

**Fig 4:**
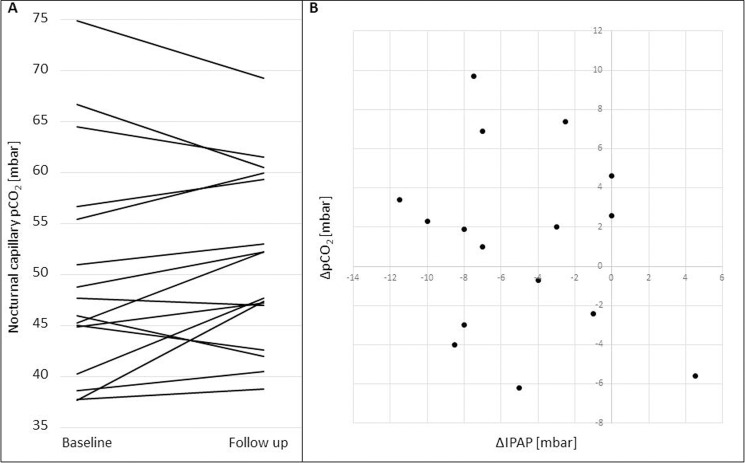
pCO_2_ values at baseline and follow-up and correlation of ΔpCO_2_ with *ΔIPAP*. A. Each line represents an early morning pCO_2_ value for a single patient at baseline and follow up. B. Scatter plot illustrating the correlation of ΔpCO_2_ (pCO_2_ at follow-up—pCO_2_ at baseline) and ΔIPAP (IPAP at follow-up—IPAP at baseline).

**Fig 5:**
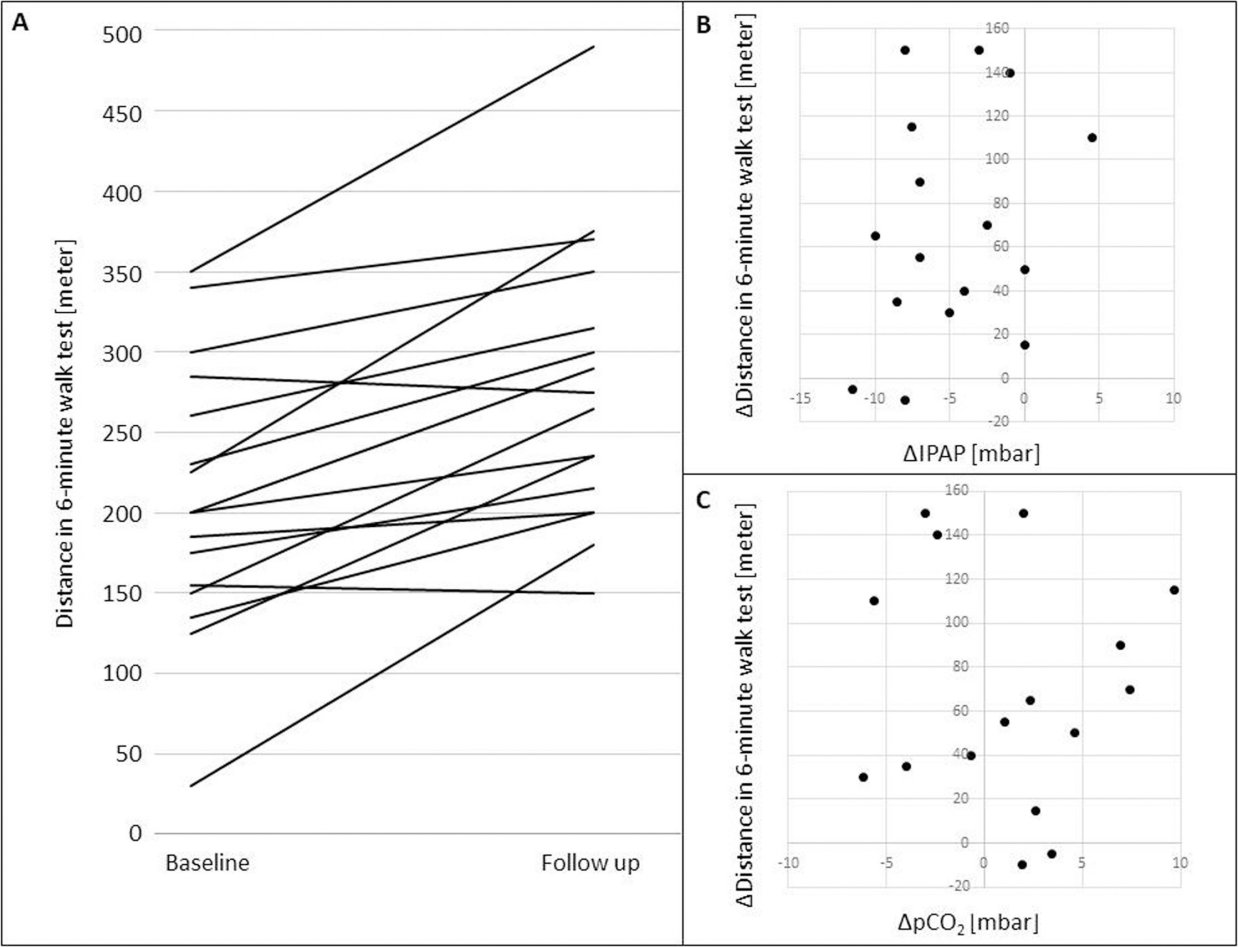
6MWT distances at baseline and follow-up *and correlations of* Δ6MWT *with ΔIPAP and ΔpCO*_*2*_. A. Walking distances in the 6MWT at baseline and at follow-up. Each line represents a patient. B. Scatter plot illustrating the correlation of Δ6MWT (6MWT at follow-up– 6MWT at baseline) and *ΔIPAP* (IPAP at follow-up—IPAP at baseline). C. Scatter plot illustrating the correlation of Δ6MWT (6MWT at follow.up– 6MWT at baseline) and *ΔpCO*_*2*_ (pCO_2_ at follow-up—pCO_2_ at baseline).
